# Can there be blood units of high and low quality?

**DOI:** 10.4103/0973-6247.45254

**Published:** 2009-01

**Authors:** N. Choudhury

**Affiliations:** *Medical Director, Prathama Blood Center, Behind Jivraj Mehta Hopital, Vasna, Ahmedabad-380 007, India*

Quality in blood transfusion services (BTS) is an important / concern for all health care professionals. Transfusion Medicine failed to draw attention from clinical specialty before outbreak of AIDS. However, due to dreaded nature of transfusion associated infections (TTI), especially of HIV, this specialty gained importance in medical community. However, quality simply does not mean TTI only. It is the overall quality implementation and management in the blood center/ bank. But how many of us in this part of the world are able/ maintain total quality management system? Unfortunately, only few of us have been able to maintain this.

At present there are more than 2455 blood banks/centers in India. And this number is increasing continuously. Do we really need this large number of blood banks/ centers in India? From functional point of view, we can broadly divide them into stand alone, hospital based and storage centers or first referral units (FRU). Though functions are/different, all these 2455 blood bank/ centers are supposed to maintain minimum quality standard. However Probably, 90% of Indian blood banks do not have the first level quality documents like quality policy and quality manual. Many blood bankers find it difficult to start writing their own quality manual. We cannot blame our colleagues for this deficiency. It is not thought about during our graduation/ post graduations nor is it made mandatory by/ Drugs and Cosmetic Acts (1945 and subsequently modified). But there are second level quality documents like standard operation procedures (SOP) and third level documents like various registers, labels and forms in all blood banks, / where these documents are mandatory from the regulatory point of view. We do not want to comment on adequacy of SOPs maintained by majority of blood banks/ centers. Many a time it is copied from some other blood banks SOPs or from standard books. Though the SOPs are supposed to be displayed in work areas, the document files remains under the closed custody of the blood bank in charge.

The situation is gradually improving in few states due to initiatives taken by National Aids Control Organization (NACO) and State Blood Transfusion Councils. They are organizing different symposiums/CME to implement systems. But is it enough to change the BTS in India? We feel that it is an up hill task. The first obstruction is the sheer large number of blood banks. Do we really require 2455 blood banks/ centers in India? India is a big country with more than 1.2 billion population. Still this number seems to be too large. We need some type of centralization. I strongly feel that we need probably about 525 central or well established Regional Blood Transfusion Centers (RBTC). This number roughly corroborates with the number of parliamentary constituencies in India. On the other hand, it also conforms to the standard of one RBTC in every 20,00,000 population. Government of India has already started initiatives to commission Metropolitan blood banks in four metros and to start state of blood banks in various states. Metropolitan blood banks should act as a technical and referral hub in four corners in India. During this selection, good performing blood banks should be selected as RBTC. There should not be any bias like government, non government organization (NGO), Red Cross Society or private sectors. All should follow quality standard, ethical standard, pricing and government regulations as and when it comes.

What is happening in different parts of the country? We all know that we blood bankers are routinely collecting blood and distributing blood/ components as medicine to our patients. As we are working in manufacturing units, we are coming under Drugs and Cosmetics Act. Different blood banks/ centers maintain different standard. As a result, blood and components going to patients are also of different quality as per the manufacturing standards. This is the reason we keep on getting news of different transfusion related incidents like wrong blood transfusion, infection positive blood transfusion or any other reason due to failure of quality system. These quality system failures may sometimes be accidental or otherwise. Why we would like to call it deliberate/otherwise? It is evident that many a times, we try to short cut or avoid things which are not convenient for our working. The simple example is hemoglobin testing in pre donation area. All of us can quote many instances where this simple test is not done. How this simple test is going to compromise the quality system if this is not preformed? We collect blood from donors with sub-standard hemoglobin which compromises with donor health. On the other hand, after transfusing this unit with low hemoglobin, the patient will not have desired rise of hemoglobin/hematocrit.

These types of compromises in quality standards are/usually observed in blood bank/ centers may be due to apathy or lack of knowledge; due to non adherence to quality standard; due to profit making motive by cutting cost. Whatever may be the reason, the people of India must get ‘blood and blood components of highest quality in sufficient quantity’ whenever needed. It is the responsibility of Transfusion Medicine fraternity and also the every Blood Transfusion Councils in India.

To remove this imbalance, The Food and Drug Control Authority (FDCA) also has significant responsibility. They are the custodians for the/ implementation of Drugs and Cosmetic Act. These types of omission and commissions are supposed to be detected, corrected and re-coursed by FDCA at State and Central level. However, it is not observed routinely. This department has their own limitations in terms of man power at field level. Another welcome move was to start the National Blood Authority in India which could implement uniform developments of all blood banks/ centers including centralizations and implementation of quality standards. However, the future of this Authority is uncertain as we have not heard about any progress in this regard

What we may try to do bring uniform quality standard to most of the blood banks. Most important issue is to bring awareness that it is important to maintain quality at any cost and management commitment is a must. It is to be initiated and implemented by national and all state blood transfusion councils. Many a times it is argued by blood bankers (both in public and charitable sectors that they provide blood free of charge at any minimal cost. Even there are competitions among charitable organizations about how low they can keep the price! We always advocate that there must be a cost recovery system in all blood banks. There is no reason why we should supply blood units without any service charges (or at minimum) when the person gets reimbursement from insurance companies, employers or if the person can afford to pay. We need a special cell in all the state councils comprising of regulatory person and a technical expert. There may be about 6-10 such committees in a state depending upon the size of the state. The Joint (or Deputy) Director, Blood Safety may coordinate activities. We should remember that this will be a fact finding mission and not a fault finding mission. The aim of these planned visits will be to improve working of the all blood banks. The inspection results should be constantly evaluated and corrective actions should be taken at appropriate level.

Another way to bring uniformity in standards is to develop awareness about accreditation. Getting accreditation is voluntary however; sensitization for this process will provide gap analysis of individual blood banks. Blood banks will come to know about their deficiencies in first/ second/ third level documents and also about deficiencies in process control. External quality assurance program (EQAS) will help to bring uniformity in quality standrad. It will help us to bring performance gap of various blood banks to the fore front. We are feeling a constant need for an EQAS program in India. There is good news that one EQAS program has been started with the support from the National Accreditation Board for Hospitals and Healthcare providers (NABH) for Indian blood banks. Another initiative blood banks/ centers can initiate is to start inter laboratory assessment scheme. It can be easily implemented among a group of blood banks in a city/ town where same sample can be blinded and distributed for testing and subsequently results can be evaluated for comparison.

In continuation on the title of this Editorial, one Ethical question arises in relation to NAT testing and supply of blood units. We all know that NAT testing is expensive and the service charge of the unit increases to two to three folds. If we go ahead for NAT testing, are we supposed to maintain two types of blood inventory i.e. NAT tested and untested? Because, both will carry different price tags. That means, rich people will opt for NAT tested and poor people will have to go for NAT untested blood? Probably, we can discuss this ethical issue sometime later.

If we look around South East Asia Region (SEAR), there are 11 countries as per norms of World Health Organization. They are Bhutan, Bangladesh, DPR Korea, India, Mayanmar, Maldevis, Nepal, Thailand, Timor Leste and Sri Lanka. Twenty five percent of world population resides in this part of the world and about 30% of disease burden is evident in this region. The blood transfusion services in member countries are in various levels of development. The estimated blood requirement in SEAR is about 15 million; however, only little more then 9 million units are collected per annum. There is a perennial shortage of blood in almost all member countries. Moreover, blood collection from voluntary non-remunerated blood donor (VNRBD) is different in different countries. The percentage of VNRBD varies from 40% to 93% in member countries. Blood is collected predominantly from family replacement donors. In Bangladesh, blood is collected from paid blood donors also. However, due to close proximity and similar ethnic and cultural relations, Pakistan and Afghanistan are included in other trans-national organizations like South Asian Association of Regional Cooperation (SAARC). The situations of BTS in these two countries are no better than others.

Quality standard: Blood transfusion services in member countries differ in terms of quality. In few countries, transfusion services are centrally coordinated like in Thailand and Sri Lanka. In other countries like India and Bangladesh, it is highly fragmented. From operational point of view, blood centers are operated by different managements like goverent, Red Cross Societies, not for profit organizations and profit making blood banks. Except few countries, regulatory mechanism is not well orchestrated.

Apart from insufficient number of VNRBD, testing of blood units for transfusion transmitted infections (TTI) is a major concern. This part of the world is medium endemic for hepatitis B and C virus. The prevalence of HIV infection is near about 1% in different countries. The area of concern is the quality of TTI testing and types of testing kits used. WHO has put lots of efforts to improve in these two areas and reasonable success has been achieved. More coordinated efforts are required to improve the quality standard in BTS. One more area of concern is blood component production and rational use of blood by clinicians. Blood component production is gradually going up but the number is not encouraging.

We as a collective group, should suggest above measures to bring up quality of our blood banks/ centers so that the products they produce is of same quality. There is no place for good and bad blood/components for needy patients. They must get high quality blood and components ffrm all licensed blood banks. If any blood bank fails to do so, they are supposed to close down their activities immediately. Let us put forward this to our national authority and or regulatory bodies for proper implementation of quality standards. As Transfusion Medicine experts, we have primary responsibility and we must guide national authority/ regulatory bodies to implement uniform quality in our blood banks/ centers.

